# A Generative Image Inpainting Model Based on Edge and Feature Self-Arrangement Constraints

**DOI:** 10.1155/2022/5904043

**Published:** 2022-10-12

**Authors:** Fan Yao, Yanli Chu

**Affiliations:** ^1^College of Information Engineering, Xizang Minzu University, Xian Yang, Shaanxi, China; ^2^College of Equipment Management and Support, Engineering University of PAP, Xi'an 710086, China

## Abstract

At present, the image inpainting method based on deep learning has achieved a better inpainting effect than traditional methods, but the inpainting results still have problems such as local structure disorder and blurred texture when the images involving a large defect area are processed. This paper proposes a second-order generative image inpainting model based on edge and feature self-arrangement constraints. The model consists of two parts: edge repair network and image repair network. Based on the self-encoder, the edge repair network generates the edges in the defect area according to the known information of the image and improves the edge repair effect by minimizing the adversarial loss and feature matching loss. The image inpainting network fills the defect area with the edge repair result as a priori condition. On the basis of U-Net, the feature self-arrangement module (FSM) is proposed to reconstruct the coding features of a specific scale, and the reconstructed feature skips to connect the decoding layer of the same scale, and it is fused with the upper layer underlying features for decoding. Meanwhile, guide loss, adversarial loss, and reconstruction loss are introduced to narrow the difference between the repaired image and the original image. The experimental results show that the inpainting results of the proposed model have stronger structural connectivity and clearer textures and the performance of PSNR, SSIM, and mean L1 loss in the Celeba, Facade, and Places2 is better than other inpainting methods, indicating that the algorithm can produce an inpainting effect with highly connected structure, reasonable semantics, and fine details.

## 1. Introduction

Image inpainting can synthesize visually realistic and semantically correct content for the missing area by using the prior information in the missing image or image training data. It is a crucial research direction in the domain of computer vision, which can be used in a variety of application scenarios, for instance, target removal, damaged or occluded area inpainting, etc.

According to different utilization characteristics, current inpainting strategies can be classified into two types: nonsemantic inpainting and semantic inpainting. Nonsemantic inpainting is a traditional image inpainting method that gradually fills the pixel information of the nondefective region of the image into the defective area through the diffusion or pixel block matching mechanism, which does not cover the generation and completion of semantic targets but only focuses on the connection and duplication and the filling of local structure and texture information. Semantic inpainting is based on deep learning. Through learning massive training data, the deep model constructs the complete mapping relationship from the damaged image to the repaired image based on the high-level semantic information extracted from the broken image and can generate semantic targets that do not exist in the background area of the broken image [1], achieving great performance in large-scale defect work that cannot be completed by traditional methods.

The current mainstream deep models all adopt generative adversarial networks (GANs) [2]. Through a continuous adversarial game between the generator and the discriminator, the repaired image quality is gradually improved. Pathak et al. [3] applied deep learning to the field of image restoration for the first time and proposed a deep network based on an autoencoder structure. By mapping the broken image features to the low-dimensional feature space by the encoder, the output signal is reconstructed with deconvolution, and the 64 × 64 rectangular defect area with the resolution of 128 × 128 image center can be repaired. However, there are obvious traces in the inpainting results. Based on the research of Pathak et al. [3], Iizuka et al. [4] used dilated convolution [5] to replace the ordinary convolution in the fully connected layer to enhance the receptive field and introduced a local discriminator to enhance the quality of details of the generated content. Finally, it is able to repair rectangular defect areas of any size. However, the model requires postprocessing similar to Poisson fusion [6] to reduce inpainting traces, and the inpainting results are significantly degraded when dealing with irregular defect area. Yu et al. [7] divided the inpainting into two stages, including rough estimation of damaged images and introducing an attention mechanism to further refine the rough inpainting result, but the inpainting effect was significantly reduced when dealing with the irregular broken area. For the poor performance of deep model when processing irregular defect area, Liu et al. [8] proposed a special convolution method and applied it in the U-Net [9] architecture, which can fill the defect area by only using the known pixel information and effectively reducing inpainting traces and chromatic aberration. Zeng et al. [10] put forward a pyramid context encoder network, which reconstructed the encoding features of each layer to constrain the decoder, obtaining high-quality inpainting results. However, there was a problem of structure disorder when repairing irregular broken images with the complex structure.

In general, the current mainstream depth model is able to generate semantically plausible content in defect regions, but there are problems of local pixel discontinuity, structure disorder, and texture blur when repairing large-area irregular broken images. For these problems, the image inpainting is decomposed into two parts in this paper: edge repair and image inpainting, and a second-order image inpainting network is proposed, which is composed of two parts: edge repair network and image repair network. First, the edge repair network extends the edge in the defect area according to the pixel information and edge information around the defect area to obtain the edge repair map and then guides the image inpainting with the edge repair map as a priori condition. In order to improve the contextual and semantic consistency of potential features at all levels in the process of image inpainting and reduce the structural features loss, U-NET network architecture is adopted to transfer the coding features at all levels to the corresponding decoding layer through skip connection, using the rich structural information and texture features in the low-level features as much as possible. Meanwhile, the feature self-arrangement module (FSM) is proposed to reconstruct the specified coding features and fuse them with the potential features and coding features of corresponding scales for the next step of decoding so as to restrict the generator to improve the final inpainting effect.

Overall, the contribution of this paper is as follows:

1. *The edge repair network is proposed*. The edge repair network generates edges in the defect area according to the high-frequency information features of the background area of the broken image, repairs the overall semantic contour of the image, perfects the local structure details, and completes the edge repair.
2. *The feature self-arrangement module (FSM) is proposed*. The feature self-arrangement module refills reasonable information in defective areas of latent features according to the correlation between the pixel block in the coded feature background area and the pixel block in the corresponding decoded feature defect area to effectively reconstruct the decoded feature.
3. *An image inpainting network is proposed*. The image inpainting network fills the pixel with output of the edge repair network as a priori condition and restricts the decoder by introducing a feature self-arrangement module on the basis of U-Net. Combining the influence of edge information and reconstruction features, high-quality repair results with complete semantics, connected structure, and clear texture can be obtained by using the image inpainting network.

## 2. Related Work

Traditional repair strategies mainly use low-level nonsemantic features in the background area of the defect image to fill in the defect area. The diffusion-based method [11–13] spreads the pixel information around the defect area from the outside region to the inside region, which can only fill in small missing areas similar to scratches and ink dots. The patch matching-based method [14, 15] can be applied into more image inpainting. The method based on pixel block matching fills the pixel information in the defect area sequentially by calculating the similarity between the boundary of the defect area and the pixel block of the background area. The traditional restoration methods only use the existing pixel information of the defect image to carry out diffusion or weighted copying, which lacks the high-level semantic understanding of the image and cannot generate the content with structural connectivity and clear texture when faced with the repair work with a complex structure and large defect area [16].

The image repair strategy based on deep learning learns high-level semantic representation from large-scale data, which greatly improves the inpainting effect. However, early convolutional neural network-based methods [6, 7] cannot handle structure and texture information separately and cannot effectively use context information to reconstruct missing content. Therefore, these methods often produce inpainting results with noise or texture artifacts when dealing with irregular defects. In order to solve these problems, many scholars conducted research from different perspectives. Liu et al. [17] divided the inpainting into two stages: rough estimation and refinement, and they proposed a coherent semantic attention layer that predicted missing content by modeling the correlation between semantic features. It is embedded in the encoder of the image refinement network to improve the inpainting ability of image details, but this method is more time-consuming and it lacks the influence of advanced context information in the attention operation. Sagong et al. [18] simplified the coarse-to-fine second-order repair network to a single-level codec network containing a contextual attention module, greatly reducing training time and computing resources and achieving high-quality inpainting results. The model proposed by Liu et al. [19] treated the deep and shallow convolution features as the structural features and texture features of the input image separately for inpainting. The two types of repaired features are balanced, fused, and transferred to the decoding layer of each scale to constrain the generated network. The semantic target with complete outline and clear texture in the large-area irregular defect area is effectively reconstructed [20].

Aiming at the above problems, this paper proposes a second-order image inpainting model that combines edge guidance and feature self-arrangement constraints. The edge repair map generated by the edge repair network ensures the structural connectivity of the final inpainting result; the feature self-arrangement module fills in the effective pixel information in the defective area of the feature level; and the skip connection fuses the coding features of each level and the FSM reconstruction features with the corresponding decoding features to guide the decoding of the next layer. The method makes full use of the context feature information on both sides of the U-Net architecture “bottleneck,” effectively reducing the blocked feature propagation in the decoding process. The image inpainting network can finally generate content with reasonable semantics, highly connected structure, and exquisite details.

## 3. Generative Image Inpainting Model

### 3.1. Model Framework

**Figure 1:**
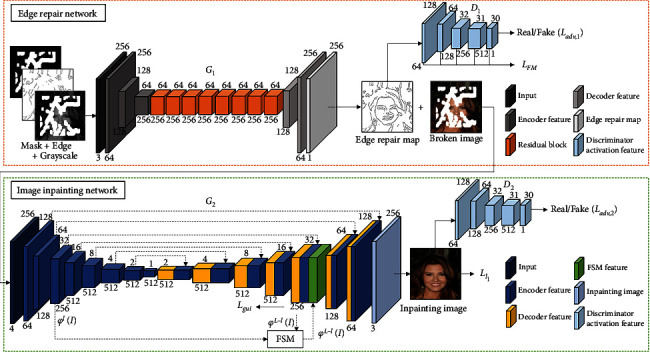
Generative image inpainting model framework.

The proposed second-order image inpainting model includes the edge repair network and the image inpainting network. The edge repair network adopts a generative confrontation network structure, which is classified into a generator *G*_1_ and a discriminator *D*_1_. The image inpainting network includes a generator *G*_2_ and a discriminator *D*_2_. The edge repair network generates a reasonable edge contour in the defect area according to the gray value of the pixels around the broken image and the edge information of the undefected area; taking the edge generation map as a priori condition, the image inpainting network outputs all levels of coding features to the corresponding decoding layer combined with skip connections and fuses with the corresponding potential features for layer decoding, which effectively uses the context information in the encoding and decoding process, reducing the information loss during feature propagation; taking the output of the specified coding layer and the underlying features of the corresponding decoding layer as input, the feature self-arrangement module fills the defect part at the image feature level to obtain the FSM reconstruction feature map. Then it is fused with the underlying features and coding features of the corresponding decoding layer for the next step decoding to improve the final inpainting effect. The specific model framework is shown in Figure 1.

### 3.2. Feature Self-Arrangement Module

For the broken image *I*, Ω is defined as the missing area and $\bar{\Omega }$ is the known area. The U-Net frame of *L*-th layer is taken as an example. *φ*^*l*^(*I*) represents the coding feature of the *l*-th layer, *φ*^*L*−*l*^(*I*) represents the decoding feature of the (*L* − *l*)-th layer, and *F*(·) represents the feature self-arrangement operation. The corresponding output FSM reconstruction feature is

(1) $$\begin{array}{c} \phi^{L-l}=F\left(\varphi^{l},\varphi^{L-l}\right)\text{.} \end{array}$$

**Figure 2:**
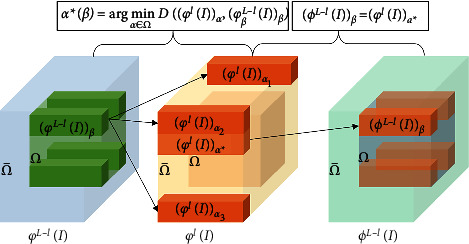
Feature self-arrangement module.

As shown in Figure 2, the feature self-arrangement module predicts *ϕ*^*L*−*l*^(*I*) through *φ*^*l*^(*I*) and *φ*^*L*−*l*^(*I*), making it closer to the output feature *φ*^*l*^(*I*_*gt*_) of the original image *I*_*gt*_ in the corresponding coding layer. For each (*φ*^*L*−*l*^(*I*))_*β*_(*β* ∈ Ω), its related nearest neighbor search in ${\left(\varphi^{l}\left(I\right)\right)}_{\alpha }\left(\alpha \in \bar{\Omega }\right)$ can be derived by the following formula:

(2) $$\begin{array}{c} \alpha^{*}\left(\beta \right)=arg\max_{\alpha \in \bar{\Omega }}\frac{\langle {\left(\varphi^{L-l}\left(I\right)\right)}_{\beta },{\left(\varphi^{l}\left(I\right)\right)}_{\alpha }\rangle }{{\left\|{\left(\varphi^{L-l}\left(I\right)\right)}_{\beta }\right\|}_{2}{\left\|{\left(\varphi^{l}\left(I\right)\right)}_{\alpha }\right\|}_{2}}\text{.} \end{array}$$

At the same time, the displacement vector of the feature self-arrangement patch block is defined as *μ*_*β*_=*α*^*∗*^(*β*) − *β*, and then (*ϕ*^*L*−*l*^(*I*))_*β*_ is predicted by copying and filling the patch block of encoding feature (*φ*^*l*^(*I*))_*α*_, that is

(3) $$\begin{array}{c} {\left(\phi^{L-l}\left(I\right)\right)}_{\beta }={\left(\varphi^{l}\left(I\right)\right)}_{\beta +\mu_{\beta }}\text{.} \end{array}$$

The feature auto-arrangement module can use the pixel block in the defective region of the decoded feature to perform matching calculation with the known pixel value of the corresponding encoding feature and refill the defective region of the decoded feature, effectively improving the content rationality at the feature level and providing a good guiding foundation for the next step of decoding.

### 3.3. Edge Repair Network

The edge repair network adopts a generative confrontation network structure, which is classified into a generator *G*_1_ and a discriminator *D*_1_. The generator adopts a self-encoder structure, and the input is composed of the mask, the gray image of the broken image, and the edge binary image which are used to obtain the shallow features through the encoder of subsampling twice. The shallow features are sent to the feature extraction area composed of eight residual blocks combined with expansion convolution (expansion factor is 2), and then the complete image is decoded by a decoder that is up-sampled twice. Spectral normalization is used for each layer in the generator network [21, 22]. Apart from the last layer of convolution of each residual block and the last layer of convolution of the decoder, the ReLU activation function is used after each layer of convolution. The network parameters of the edge generator are shown in Table 1.

The edge discriminator uses the Path GAN [23] architecture. Path GAN maps the input image to a matrix *X* of *N* × *N* through convolution, and each point of the matrix *X*; that is, the value of *X*_*i*,*j*_ stands for the evaluation of a small area of the input image. Finally, the average value of *X*_*i*,*j*_ is the output of the discriminator. The introduction of Path GAN can make the edge repair network attach importance to image details in training. The training process is stabilized by adding spectral normalization to each layer of the discriminator network [21]. After the first four layers are convolved, the slope parameters of Leaky ReLU are set to 0.2, and the sigmoid activation function is utilized before the discriminator outputs the final result. The network layer parameters of the edge discriminator are shown in Table 2.

### 3.4. Image Inpainting Network

Similar to the edge generation network, the image inpainting network includes a generator *G*_2_ and a discriminator *D*_2_. Skip connection is applied at each coding layer of the generator to transfer the output of each coding layer to the corresponding decoding layer, and it is fused with the underlying features to decode layer by layer, which takes full advantage of the context information in the encoding and decoding process to decrease the information loss during the layer-by-layer transmission of features at all levels. In addition, in order for the generator to generate content with consistent contextual semantics and rich low-level details in the decoding process, a feature self-arrangement module is proposed, which performs pixel rearrangement and fills in the defect areas with the coding features and decoding features of the same scale as input. Finally, FSM feature map with a complete semantic and structural connection can be reconstructed. This method fills reasonable information in the defect area at the feature level, playing a positive role in improving and guiding the subsequent decoding at all levels. By minimizing the introduced guidance loss, the specific decoding layer of the generator is obliged to output a feature map that conforms to the real situation as much as possible. Meanwhile, reconstruction loss and counter loss are applied to the final output image to train the network, thereby continuously improving the inpainting effect of the network.

The image repair network generator is divided into encoding and decoding. *Enc*(·) represents the encoding process, *I*_*gt*_^*brk*^ represents the broken RGB image, and *E*_*pre* *d*_ represents the edge repair map. The input of the image repair network is represented as *I*=*I*_*gt*_^*brk*^ ⊕ *E*_*pre* *d*_. The encoding features at all levels are expressed as

(4) $$\begin{array}{c} \varphi^{1}\left(I\right)=Enc\left(I\right)\text{,} \end{array}$$

(5) $$\begin{array}{c} \varphi^{2}\left(I\right)=Enc\left(\varphi^{1}\left(I\right)\right)\text{,} \end{array}$$

(6) $$\begin{array}{c} \varphi^{3}\left(I\right)=Enc\left(\varphi^{2}\left(I\right)\right)\text{,} \end{array}$$

(7) $$\begin{array}{c} \varphi^{8}\left(I\right)=Enc\left(\varphi^{7}\left(I\right)\right)\text{.} \end{array}$$

By introducing skip connections, the coding features and corresponding decoding features at each decoding layer are fused for decoding. *Dec*(·) represents the decoding operation. *F*(·) represents the feature self-arrangement operation. The decoding features at all levels are expressed as

(8) $$\begin{array}{c} \varphi^{9}\left(I\right)=Dec\left(\varphi^{8}\left(I\right)\right)\text{,} \end{array}$$

(9) $$\begin{array}{c} \varphi^{10}\left(I\right)=Dec\left(\varphi^{9}\left(I\right)⊕\varphi^{7}\left(I\right)\right)\text{,} \end{array}$$

(10) $$\begin{array}{c} \phi^{13}\left(I\right)=F\left(\varphi^{13}\left(I\right)⊕\varphi^{3}\left(I\right)\right)\text{,} \end{array}$$

(11) $$\begin{array}{c} \varphi^{14}\left(I\right)=Dec\left(\left(\varphi^{13}\left(I\right)⊕\varphi^{3}\left(I\right)\right)⊕\phi^{13}\right)\text{,} \end{array}$$

(12) $$\begin{array}{c} \varphi^{15}\left(I\right)=Dec\left(\varphi^{14}\left(I\right)⊕\varphi^{2}\left(I\right)\right)\text{.} \end{array}$$

Instance normalization is applied to each convolution layer of the image generator network except the first and last layers [24]. The slope parameters of Leaky ReLU are set to 0.2. The network parameters of the image generator are shown in Table 3.

The image discriminator *D*_2_ and the edge discriminator *D*_1_ adopt the same network architecture and parameter settings, and the generator generates content that looks like the raw image by minimizing the adversarial loss.

## 4. Loss Function

### 4.1. Overall Loss Function

The overall loss function of the proposed model is

(13) $$\begin{array}{c} L_{Gen}=L_{e\;dg\;e}+L_{img}=\lambda_{a\;dv,1}L_{a\;dv,1}+\lambda_{FM}L_{FM}+\lambda_{a\;dv,2}L_{a\;dv,2}+\lambda_{gui}L_{gui}+\lambda_{l_{1}}L_{l_{1}}\text{,} \end{array}$$

where *L*_e dg e_ is the overall loss function of the edge repair network; *L*_img_ is the overall loss function of the image repair network; *L*_a dv,1_ is the adversarial loss of the edge discriminator, which is applied to train the edge generation network; *L*_FM_ is the feature matching loss of the edge generation network; *L*_a dv,2_ is the adversarial loss of the image repair network; *L*_gui_ is the guiding loss of the output by the specific decoding layer of the constrained image repair network; *L*_*l*_1__ is the reconstruction loss of the image repair network. *λ*_a dv,1_, *λ*_FM_, *λ*_a dv,2_, *λ*_gui_, and *λ*_*l*_1__ are the corresponding weight parameters. Each loss function is described in detail below.

### 4.2. Edge Loss

*I*_gt_, *I*_gray_, and *E*_gt_, respectively, represent the original image of the input edge repair network and its grayscale image and edge binary image; in the mask *M*, the missing area is marked as 1, and the background area is marked as 0. The broken image is expressed as *I*_gt_^brk^=*I*_*gt*_⊙(1 − *M*), and the defect grayscale image is expressed as *I*_gray_^brk^=*I*_gray_⊙(1 − *M*), and the defect edge binary image is expressed as *E*_gt_^brk^=*E*_*gt*_⊙(1 − *M*); ⊙is the Hadamard product, which means that the corresponding elements of the matrix are multiplied. *G*_1_(·) stands for the operation of the edge generator, and *D*_1_(·) stands for the operation of the edge discriminator, then the edge repair map is expressed as *E*_pre d_=*G*_1_(*I*_gray_^brk^, *E*_gt_^brk^, *M*).

The adversarial loss *L*_a dv,1_ [1] is defined as follows, which is used to train the edge repair network:

(14) $$\begin{array}{c} L_{a\;dv,1}=E_{\left(E_{gt},I_{gray}\right)}\log\;\left[D\left(E_{gt},I_{gray}\right)\right]+E_{I_{gray}}\log\;\left[1-D\left(E_{pre\;d},I_{gray}\right)\right]\text{.} \end{array}$$

The feature matching loss is introduced [25]. By comparing the activation features of the edge repair image and the original image edge in the middle layer of the discriminator, the generator is forced to produce more realistic and reasonable results to stabilize the training process. *L* represents the number of convolutional layers of *D*_1_, *N*_*i*_ represents the number of elements in the *i*-th active layer of *D*_1_, and *D*_1_^(*i*)^ represents the activation feature map of the *i*-th layer in the discriminator. By comparing the activation maps of the middle layer of the discriminator, the generator is forced to produce results closer to the raw image to stabilize the training process. The feature matching loss is as follows:

(15) $$\begin{array}{c} L_{FM}=E\left[\overset{L}{\underset{i=1}{\sum }}\frac{1}{N_{i}}{\left\|D_{1}^{\left(i\right)}\left(E_{gt}\right)-D_{1}^{\left(i\right)}\left(E_{pre\;d}\right)\right\|}_{1}\right]\text{.} \end{array}$$

The overall loss function of the edge generation network is

(16) $$\begin{array}{c} L_{e\;dg\;e}=\lambda_{a\;dv,1}L_{a\;dv,1}+\lambda_{FM}L_{FM}\text{,} \end{array}$$

where *λ*_a dv,1_ and *λ*_FM_ are the weight parameters. *λ*_a dv,1_=1, *λ*_FM_=15.

### 4.3. Image Inpainting Loss

*G*_2_(·) stands for the operation of the image generator, and *D*_2_(·) stands for the operation of the image discriminator; the generated image is expressed as *I*_*p*re d_=*G*_2_(*I*_gt_^brk^, *E*_pre d_, *M*). The final output of the entire inpainting network is the fused image, defined as *I*_gt_^comp^=*I*_gt_^brk^+*I*_pre d_⊙*M*.

The adversarial loss [2] *λ*_a dv,2_ is introduced, which is used to train the image inpainting network.

(17) $$\begin{array}{c} L_{a\;dv,2}=E_{\left(I_{gt},I_{pre\;d}\right)}\log\;\left[D_{2}\left(I_{gt},E_{pre\;d}\right)\right]+E_{E_{pre\;d}}\log\;\left[1-D_{2}\left(I_{pre\;d},E_{pre\;d}\right)\right]\text{.} \end{array}$$

In the decoding process, in order to retain more of the raw information of the image, the guidance loss is introduced to judge the output feature of the *L* − *l*-th layer, so as to reduce the difference between the output *φ*^*L*−*l*^(*I*) and *φ*^*l*^(*I*_*gt*_) of the decoding layer and enhance the decoding capability of the model. The guidance loss is as follows:

(18) $$\begin{array}{c} L_{gui}={\underset{\beta \in \Omega }{\sum }\left\|{\left(\varphi^{L-l}\left(I\right)\right)}_{\beta }-{\left(\varphi^{l}\left(I_{gt}\right)\right)}_{\beta }\right\|}_{2}^{2}\text{.} \end{array}$$

The reconstruction loss is introduced to evaluate the final repaired image, so as to make the content generated by the image generator closer to the real image. The reconstruction loss is as follows:

(19) $$\begin{array}{c} L_{l_{1}}={\left\|I_{pre\;d}-I_{gt}\right\|}_{1}\text{.} \end{array}$$

The overall loss function of this module is

(20) $$\begin{array}{c} L_{img}=\lambda_{a\;dv,2}L_{a\;dv,2}+\lambda_{gui}L_{gui}+\lambda_{l_{1}}L_{l_{1}}\text{,} \end{array}$$

where *λ*_a dv,2_=0.1, *λ*_gui_=0.01, and *λ*_*l*_1__=0.5.

## 5. Experimental Comparison and Analysis

### 5.1. Experimental Set-Up

This network runs under the Windows10 platform, Intel Xeon E5 is used by the CPU, NVIDIA RTX 2070 is used by the GPU, the GPU memory is 8G; the depth learning development frame is PyTorch, and the CUDA installed version is V10.0. Two networks are trained by using the mask set offered by Liu et al. [8], and the image size of the input network is uniformly adjusted to 256 × 256, and the mask required for each image was randomly selected from the mask library published by Liu et al. [8]. The mask library contained 12,000 irregular masks, and the mask rate was divided into six categories: (0, 0.1], (0.1, 0.2], [(0.3, 0.4, 0.2, 0.3]], (0.4, 0.5], and (0.5, 0.6]. When the edge repair network is trained, the batchsize is 8, the Adam optimizer with parameter: beta1 = 0, beta2 = 0.9 is used for optimization. The ratio of the learning rate of the generator to the discriminator is 0.1. At the beginning of network training, the learning rate uses 1 × 10^−4^, and when the loss tends to be stable, the learning rate is changed to 1 × 10^−5^, and then training until convergence is realized. When the image inpainting network is trained, batchsize is set to 4, and the remaining training parameters and strategies are consistent with the edge repair network.

Experiments are conducted on three datasets with different styles: Celeba [26], Façade [27], and Places2 [28], and GL [4] PCONV [8], and PEN-Net [10] are selected to compare with the proposed model.

### 5.2. Wavelet Filterbank Theory

#### 5.2.1. Qualitative Comparison

**Figure 3:**
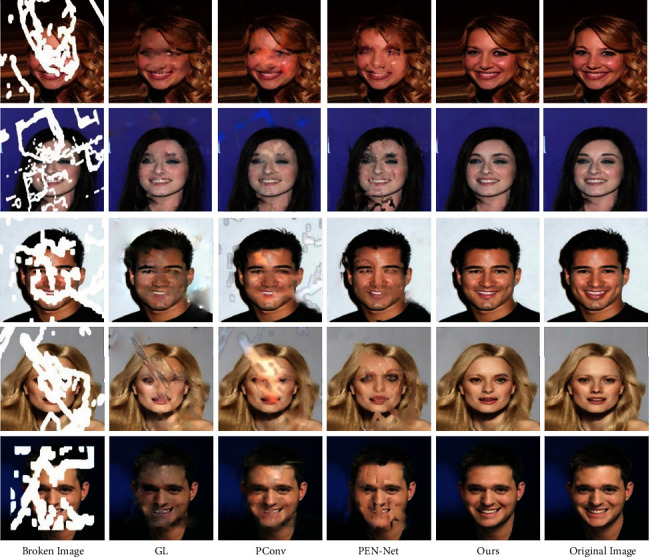
Comparison results on the Celeba.

GL performs poorly in color consistency and structural connectivity when repairing irregular and defective face images. Although two discriminators are used to evaluate the overall semantic information and local detailed features of the inpainting results, GL does not perform targeted filling and inpainting of the defect area at the feature level, and the performance of GL in the large-area irregular broken image inpainting is inferior to that in regular rectangular area. The content generated by PConv in the defect area is close to the original image in color, but there is an obvious blur. Moreover, due to the lack of the guidance of the marginal prior conditions, there is structural disorder in the inpainting result. PEN-Net is not stable enough to repair such irregular face images. It can generate semantic targets (such as eyes) in the defect area that are not found in the surrounding background area, but lacking prerequisite guidance for the edge results in the generated content with reasonable semantics but disorderly location, which affects the inpainting effect. Taking the edge repair map as the guiding factor, through the flexible combination of the feature self-arrangement module and the skip connection, the context feature information is fully used, and then the clear and reasonable content can be obtained. The specific inpainting effect is shown in Figure 3.

**Figure 4:**
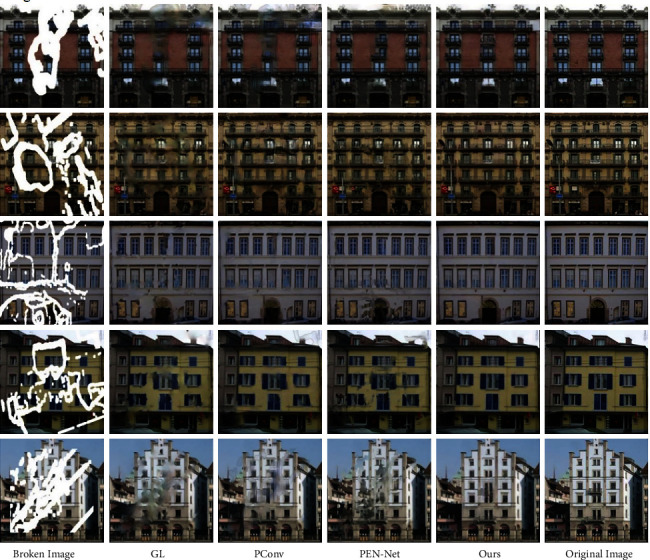
Comparison results on the Facade.

The inpainting result of the image with the complex structure such as the exterior wall of the building by GL is relatively blurred. PConv can generate content with similar structure and color to the raw image in the defect area, but there are still obvious artifacts. The attention shift mechanism applied by PEN-Net can generate pixel content similar to the original image at the defect location, and the artifact is significantly improved. However, due to the lack of guidance from the edge, the structure connectivity of the generated content is insufficient. When the algorithm in this paper repairs such irregular broken images with complex structures, the edge generation map repaired by the edge repair network plays a very important guiding role. Because the edge repair network can generate a well-structured edge in the defect area, the final inpainting result avoids structural disorder. The specific inpainting effect is shown in Figure 4.

**Figure 5:**
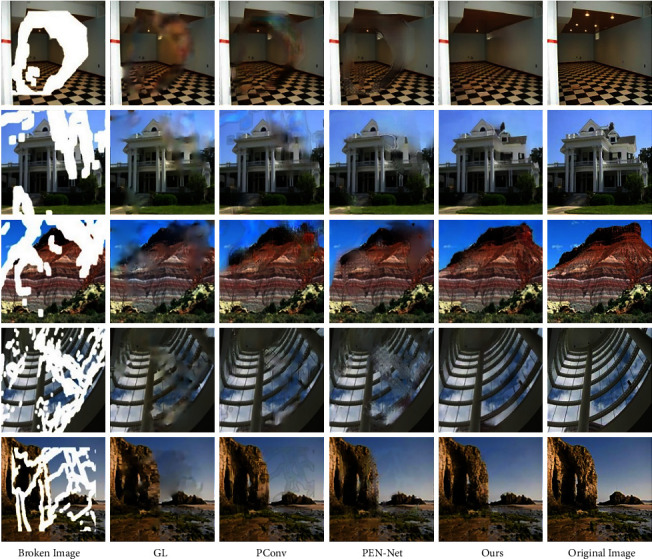
Comparison results on the Places2.

Both GL and PCONV have different degrees of artifacts in the inpainting of natural images. Pen-Net performs well in repairing solid color background areas, but the model has poor performance when processing natural image texture synthesis, resulting in color distortion and serious blurring, which generates content with poor structural connectivity and unreasonable color content. The edge repair network in this paper generates reasonable structural information in the broken area to ensure the structural connectivity of the final inpainting effect. Through skip connection and feature matching rearrangement network, the potential feature information at all levels is fully utilized to ensure the semantic coherence and color rationality of the final inpainting effect. The specific inpainting effect is shown in Figure 5.

#### 5.2.2. Quantitative Comparison

In order to objectively compare the repair effects of this model and other algorithms, mean L1 loss, peak signal-to-noise ratio (PSNR), and structural similarity index measurement (SSIM) are adopted to evaluate the inpainting results. The mean L1 loss is used to compare the L1 distance between the raw image and the repaired image. PSNR focuses on the difference between image pixels, and the SSIM is selected to compare the difference between the two images in brightness, contrast, and structure. The quantitative comparison results are shown in Table 4. The lower the value of mean L1 loss is, the better. The higher the value of PSNR and SSIM are, the better. The method with the optimal inpainting effect under different mask rates has been shown in bold († indicates that the lower the value is, the better, and ¶ indicates that the higher the value is, the better).

### 5.3. Model Validity Analysis

#### 5.3.1. Validity Analysis of Edge Repair Network

**Figure 6:**
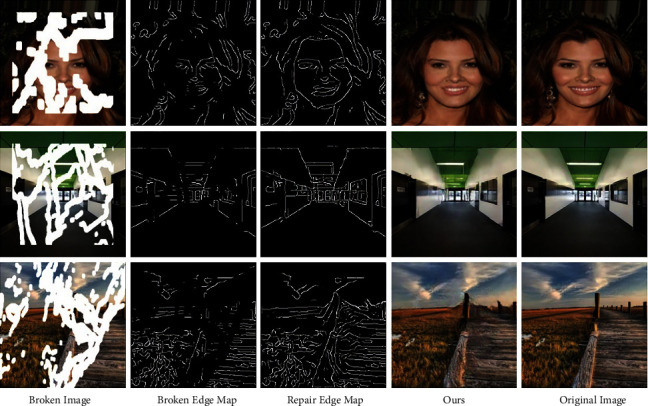
Edge repair network validity test.

Under the condition of given gray information and edge information around the broken area, the edge repair network can extend the edge in the broken area and generate a partially closed and coherent semantic contour. The comparison of the broken edge map and the edge repair map in Figure 6 shows that the edge repair network can connect the two ends of the broken structure in the broken area, which fully indicates that the edge repair network can accurately reconstruct high-frequency structural information. At the same time, the analysis of the repaired image by the edge repair map shows that the semantic target structure in the broken area is highly consistent with that of the edge generated map in the broken area, which proves the validity of image inpainting with the edge repair map as a prior condition.

#### 5.3.2. Validity Analysis of Edge Prior Conditions

**Figure 7:**
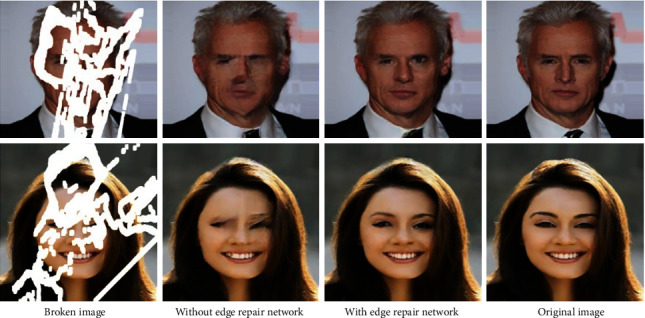
Validity test of edge prior conditions.

With the edge repair map as a priori condition, the model can finally generate semantically coherent content in the broken area. Without the edge repair map as a guide condition, the model cannot generate content with reasonable semantics and connected structure. It fully illustrates the important role of the edge generated map in the image inpainting to insure the structural connectivity and semantic coherence of the inpainting effect, as shown in Figure 7.

#### 5.3.3. FSM Feature Validity Analysis

**Figure 8:**
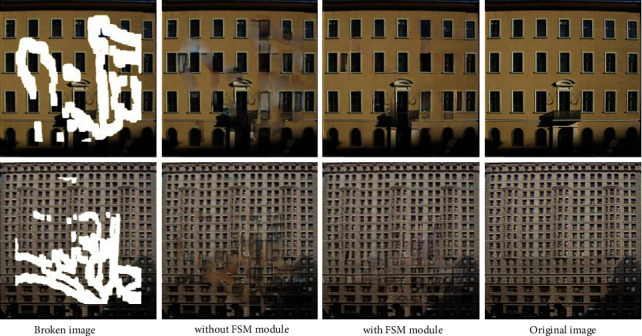
FSM feature validity test.

Without FSM feature guiding inpainting, the final inpainting result generated in this paper is quite distinct from the background region in color, which does not conform to the human visual characteristics; when the FSM feature guidance is introduced in the decoding process, the content generated by the model in the broken area is highly consistent with the background area in color, and the texture blur in the broken area is effectively reduced, indicating that the introduction of FSM features in the decoding process enables the model to generate content with more reasonable color and clearer details, as shown in Figure 8.

## 6. Conclusions

A generative image inpainting model combining edge and feature self-arrangement module is proposed. The edge repair network can effectively reconstruct structural information in the broken area and output a highly connected and semantically coherent edge repair map; the image inpainting network uses edge generation map as prior conditions and fuses the encoding features at all levels with the corresponding decoding features to decode layer by layer through skip connection, which effectively utilizes the context feature information in the process of encoding and decoding, avoiding the loss of part of semantic information and local details when the context features are transmitted among convolutional layers; meanwhile, a feature self-arrangement module is proposed to fill the broken area with effective information at the feature level, and it fuses the reconstruction features into the decoding process of the corresponding layer to restrain the subsequent decoding. Combined with the influence of the above three aspects, the model of this article can eventually generate the content with the same semantics, valid structure, and clear texture features as the original image.

## Figures and Tables

**Table 1:** The network parameters of the edge generator.

Layer	Inputs (*H* × *W* × C)	Kernel size	Stride	Padding	Dilation	Activation function	Outputs (*H* × *W* × C)
ReflectionPad	256 × 256 × 3	—	—	3	—	—	262 × 262 × 3
Conv	262 × 262 × 3	7 × 7	1	0	1	ReLU	256 × 256 × 64
Conv	256 × 256 × 64	4 × 4	2	1	1	ReLU	128 × 128 × 128
Conv	128 × 128 × 128	4 × 4	2	1	1	ReLU	64 × 64 × 256

Residual Blocks × 8							
ReflectionPad	64 × 64 × 256	—	—	2	—	—	68 × 68 × 256
Res-conv	68 × 68 × 256	3 × 3	1	0	2	ReLU	64 × 64 × 256
ReflectionPad	64 × 64 × 256	—	—	1	—	—	66 × 66 × 256
Res-conv	66 × 66 × 256	3 × 3	1	0	1	—	64 × 64 × 256
ConvTranspose	64 × 64 × 256	4 × 4	2	1	1	ReLU	128 × 128 × 128
ConvTranspose	128 × 128 × 128	4 × 4	2	1	1	ReLU	256 × 256 × 64
ReflectionPad	256 × 256 × 64	—	—	3	—	ReLU	262 × 262 × 64
Conv	262 × 262 × 64	7 × 7	1	0	1	—	256 × 256 × 1

**Table 2:** Network parameters of the edge discriminator.

Layer	Inputs (*H* × *W* × C)	Kernel size	Stride	Padding	Dilation	Activation function	Outputs (*H* × *W* × C)
Conv	256 × 256 × 2	4 × 4	2	1	1	Leaky ReLU	128 × 128 × 64
Conv	128 × 128 × 64	4 × 4	2	1	1	Leaky ReLU	64 × 64 × 128
Conv	64 × 64 × 128	4 × 4	2	1	1	Leaky ReLU	32 × 32 × 256
Conv	32 × 32 × 256	4 × 4	1	1	1	Leaky ReLU	31 × 31 × 512
Conv	31 × 31 × 512	4 × 4	1	1	1	—	30 × 30 × 1

**Table 3:** Network parameters of the image generator.

Layer	Inputs (*H* × *W* × C)	Kernel size	Stride	Padding	Dilation	Activation function	Outputs (*H* × *W* × C)
Conv	256 × 256 × 4	4 × 4	2	1	1	—	128 × 128 × 64
Conv	128 × 128 × 64	4 × 4	2	1	1	Leaky ReLU	64 × 64 × 128
Conv	64 × 64 × 128	4 × 4	2	1	1	Leaky ReLU	32 × 32 × 256
Conv	32 × 32 × 256	4 × 4	2	1	1	Leaky ReLU	16 × 16 × 512
Conv	16 × 16 × 512	4 × 4	2	1	1	Leaky ReLU	8 × 8 × 512
Conv	8 × 8 × 512	4 × 4	2	1	1	Leaky ReLU	4 × 4 × 512
Conv	4 × 4 × 512	4 × 4	2	1	1	Leaky ReLU	2 × 2 × 512
Conv	2 × 2 × 512	4 × 4	2	1	1	Leaky ReLU	1 × 1 × 512
DeConv	1 × 1 × 512	4 × 4	2	1	1	ReLU	2 × 2 × 512
DeConv	2 × 2 × 1024	4 × 4	2	1	1	ReLU	4 × 4 × 512
DeConv	4 × 4 × 1024	4 × 4	2	1	1	ReLU	8 × 8 × 512
DeConv	8 × 8 × 1024	4 × 4	2	1	1	ReLU	16 × 16 × 512
DeConv	16 × 16 × 1024	4 × 4	2	1	1	ReLU	32 × 32 × 256
DeConv	32 × 32 × 768	4 × 4	2	1	1	ReLU	64 × 64 × 128
DeConv	64 × 64 × 256	4 × 4	2	1	1	ReLU	128 × 128 × 64
DeConv	128 × 128 × 128	4 × 4	2	1	1	Tanh	256 × 256 × 3

**Table 4:** Quantitative comparison of algorithms.

Data set	Mask rate	Mean L1 loss†				PSNR¶				SSIM¶			
		GL	PConv	PEN-Net	Ours	GL	PConv	PEN-Net	Ours	GL	PConv	PEN-Net	Ours
Celeba	10% ∼ 20%	0.035	0.031	0.026	**0.012**	25.11	26.29	27.69	**31.65**	0.853	0.885	0.912	**0.931**
	20% ∼ 30%	0.062	0.051	0.041	**0.036**	23.96	24.21	26.44	**30.21**	0.811	0.817	0.826	**0.839**
	30% ∼ 40%	0.081	0.055	0.051	**0.037**	23.64	23.85	23.86	**27.03**	0.795	0.811	0.811	**0.841**
	40% ∼ 50%	0.101	0.089	0.072	**0.065**	22.21	21.47	22.37	**26.41**	0.713	0.786	0.797	**0.823**

Facade	10% ∼ 20%	0.043	0.033	0.031	**0.019**	23.08	23.59	24.19	**32.36**	0.801	0.857	0.841	**0.943**
	20% ∼ 30%	0.047	0.073	0.045	**0.044**	23.57	23.21	23.91	**27.17**	0.774	0.863	0.861	**0.889**
	30% ∼ 40%	0.088	0.091	0.073	**0.055**	21.15	22.19	22.57	**25.33**	0.766	0.712	0.854	**0.871**
	40% ∼ 50%	0.101	0.113	0.076	**0.067**	21.67	22.31	21.34	**23.57**	0.714	0.751	0.836	**0.857**

Places2	10% ∼ 20%	0.037	0.021	0.023	**0.011**	21.61	23.61	25.33	**30.19**	0.784	0.835	0.814	**0.919**
	20% ∼ 30%	0.057	0.054	0.040	**0.031**	21.17	22.71	24.74	**27.47**	0.776	0.797	0.804	**0.814**
	30% ∼ 40%	0.051	0.058	0.053	**0.046**	20.53	22.15	23.87	**25.31**	0.751	0.779	0.789	**0.826**
	40% ∼ 50%	0.071	0.083	0.075	**0.066**	20.27	21.94	21.61	**22.92**	0.737	0.764	0.778	**0.793**

The bold values are the experimental results in this paper.

## Data Availability

The datasets analyzed during the current study are available from the corresponding author on reasonable request.
